# Prenatal Rare 16q24.1 Deletion Between Genomics and Epigenetics: A Review

**DOI:** 10.3390/genes16080873

**Published:** 2025-07-24

**Authors:** Valentina Fumini, Romina Bonora, Anna Busciglio, Francesca Cartisano, Paola Celli, Ilaria Gabbiato, Nicola Guercini, Barbara Mancini, Donatella Saccilotto, Anna Zilio, Daniela Zuccarello

**Affiliations:** Medical Genetics and Genomic Unit, San Bortolo Hospital, 36100 Vicenza, Italy; valentina.fumini@aulss8.veneto.it (V.F.); romina.bonora@aulss8.veneto.it (R.B.); anna.busciglio@aulss8.veneto.it (A.B.); francesca.cartisano@studenti.unipd.it (F.C.); paola.celli@aulss8.veneto.it (P.C.); ilaria.gabbiato@aulss8.veneto.it (I.G.); nicola.guercini@aulss8.veneto.it (N.G.); barbara.mancini@aulss8.veneto.it (B.M.); donatella.saccilotto@aulss8.veneto.it (D.S.); anna.zilio@aulss8.veneto.it (A.Z.)

**Keywords:** 16q24.1 microdeletion, *FOXF1*, alveolar capillary dysplasia with misalignment of pulmonary veins

## Abstract

Alveolar capillary dysplasia with misalignment of pulmonary veins (ACDMPV) is a rare, often fatal congenital disorder characterized by severe neonatal respiratory distress and associated with complex multisystem malformations. In approximately 90% of cases, the condition is linked to deletions or mutations affecting the *FOXF1* gene or its upstream enhancer region on chromosome 16q24.1. This review analyzes reported prenatal cases with 16q24.1 deletion involving *FOXF1*, aiming to identify recurrent sonographic features and elucidate the underlying genomic and epigenetic mechanisms. We reviewed prenatal cases reported in the literature involving deletions of the 16q24.1 region, including the *FOXF1* gene. Here, we expand the case series by reporting a fetus with increased nuchal translucency measuring 8 mm and a de novo 16q24.1 deletion. We identified nine prenatal cases with a 16q24.1 deletion, all involving the *FOXF1* gene or its enhancer region. The main ultrasound findings included increased nuchal translucency and cystic hygroma during the first trimester, and cardiac, renal, and intestinal malformations from 20 weeks of gestation onward. Prenatal diagnosis of ACDMPV based solely on ultrasound findings is challenging. In most reported cases, the pregnancy was carried to term, with the diagnosis being confirmed by post-mortem histopathological examination. In the only case in which the pregnancy was terminated at 14 weeks’ gestation, histological examination of the fetal lungs, despite them being in the early stages of development, revealed misaligned pulmonary veins in close proximity to the pulmonary arteries and bronchioles. Evidence highlights the significance of non-coding regulatory regions in the regulation of *FOXF1* expression. Differential methylation patterns, and possible contributions of parental imprinting, highlight the complexity of *FOXF1* regulation. Early detection through array comparative genomic hybridization (array CGH) or next-generation sequencing to identify point mutations in the *FOXF1* gene, combined with increased awareness of ultrasound markers suggestive of the condition, could improve the accuracy of prenatal diagnosis and genetic counseling. Further research into the epigenetic regulation of *FOXF1* is crucial for refining recurrence risk estimates and improving genetic counseling practices.

## 1. Introduction

Alveolar capillary dysplasia with pulmonary vein misalignment (ACDMPV, MIM 265380) is a rare and fatal congenital disorder of lung development. This condition was first described in 1981 by Janney et al. [1], in a patient with a failure in the formation and ingrowth of alveolar capillaries. Since then, over 200 cases have been reported. A slight male predominance (60%) was initially observed [2,3], although no supporting evidence has emerged from subsequent studies.

Symptoms of ACDMPV include severe respiratory distress caused by pulmonary arterial hypertension and insufficient oxygen uptake, typically manifesting within the first 24–48 h of life in neonates born at full term with a normal Apgar score. In more than 80% of cases, ACDMPV is associated with other manifestations affecting various systems, such as the cardiovascular system (e.g., hypoplastic left heart, coarctation of the aortic arch), the gastrointestinal system (with malrotation being the most common sign), and the genitourinary system [4].

Regardless of co-morbidities, mortality is high, with most patients dying due to hypoxemic respiratory failure that is resistant to therapy within a few days to a week after disease onset or due to severe irreversible pulmonary arterial hypertension. Some patients with a milder form of ACDMPV exhibit longer survival and, in these cases, lung transplantation may be a potential option [5].

Histologically, the lung tissue in ACDMPV displays a misalignment of the pulmonary veins, characterized by their abnormal positioning in the bronchovascular bundle. Normally located within the interlobular septa, the veins are instead found outside the septa, adjacent to the pulmonary arteries. Additionally, there is medial thickening of the smooth muscle in the pulmonary arteries, leading to the formation of small peripheral arteries. Other features include hyperplasia of the alveolar epithelium, a reduction in the number of capillaries and underdevelopment of the lung lobules [2].

Histological examination is considered the gold standard for diagnosing ACDMPV and the diagnosis is typically confirmed postmortem through autopsy [2].

Heterozygous point mutations of the *FOXF1* gene or deletions involving *FOXF1* and/or the enhancer region at 16q24.1 are found in about 90% of cases of ACDMPV, suggesting that a haploinsufficiency mechanism involving this gene is responsible for the disease.

## 2. Materials and Methods

### 2.1. Review Strategy

We searched Pubmed (last assessed: March 2025) for papers describing prenatal cases with chromosome 16q24.1 deletion, focusing on reports in English and extended our search to papers mentioned in the reference lists of identified papers.

The search was performed using various combinations of the keywords: ‘prenatal microdeletion 16q24.1,’ ‘*FOXF1*,’ ‘cystic hygroma,’ and ‘prenatal misalignment of the pulmonary veins.

Identified patients from the literature were grouped according to the cytogenetic location of their deletions and reported symptoms were collected and counted for each group. All genetic coordinates are given in genome build hg19.

### 2.2. Array-CGH Analysis (Only Case 9)

Prenatal diagnosis of the fetus in case 9 was performed using routine diagnostic procedures. Informed consent signed by the parents was provided. DNAs were isolated from chorionic villus (fetus) and peripheral blood (parents) samples. Array-CGH analysis was performed using CytoSure Oligo array ISCA v2 4 × 180 K OGT( Oxford Gene Technology, Oxford, UK), resolution ~80 kb and CytoSure Interpret Software v4.11. Protocols provided by the suppliers have been followed without modification. Nucleotide designations were assigned according to the GRCh37/hg19 assembly of the human genome.

The 16q24.1 region gene content has been assessed merging the bioinformatics data from UCSC Genome Browser (available online: https://genome.ucsc.edu/ accessed on 2 February 2023), DECIPHER (available online: https://www.deciphergenomics.org/ accessed on 2 February 2023), OMIM (available online: https://www.omim.org/ accessed on 2 February 2023), and gnomAD databases (available online: https://gnomad.broadinstitute.org/ accessed on 2 February 2023).

## 3. Results

### 3.1. Case 1

The first prenatal case was reported in a fetus at 18 weeks of gestation presenting with cystic hygroma, fetal hydrops, and a single umbilical artery. The family history was negative for genetic diseases, intellectual disabilities, congenital malformations, or consanguinity. A microarray analysis performed on amniotic fluid revealed a 1.1 Mb interstitial deletion in the 16q24.1 region, involving the *FOXF1* and *FOXC2* genes. Other deleted genes included *MTHFSD*, *FOXL1*, *IRF8*, *COX4I1*, and *COX4NB*, none of which are known to be associated with a specific phenotype. The pregnancy was terminated at 22 weeks of gestation. An external evaluation revealed low-set ears and soft tissue edema in the neck. Unfortunately, a histopathological examination of the lungs was not conducted [6].

### 3.2. Case 2

A fetus at 20 weeks of gestation was described presenting with echographic signs of an atrioventricular septal defect and a dilated bowel loop, likely due to upper gastrointestinal atresia. Karyotype analysis on amniotic fluid revealed a pericentric inversion of chromosome 16: 46,XX,inv(16)(p11.2q24)dn. Microarray analysis confirmed that the inversion was balanced. Using BAC clones from chromosome 16 in combination with dual-color FISH, one of the breakpoints was mapped to the q-arm, between the *IRF8* and *FOXF1* genes, at a distance of 123 to 163 kb upstream of *FOXF1*. This region is thought to contain regulatory elements essential for the expression of both genes. A female infant was born at 39 + 4 weeks of gestation with Apgar scores of 4 and 7. At birth, she exhibited mild respiratory distress. Postnatal evaluation confirmed the presence of an atrioventricular septal defect and dilated bowel loops. At 20 h of age, her respiratory condition deteriorated and she passed away at 66 h of life. Autopsy findings confirmed the atrioventricular septal defect and duodenal atresia, while lung biopsy findings were consistent with ACDMPV [7].

### 3.3. Case 3 and 4

Szafranski et al. [8] reported two fetuses; the first one showed cystic hygroma, hydronephrosis, single umbilical artery and coarctation of aorta, and 1.596 Kb deletion involving *FOXF1* and the enhancer region. The second one showed bilateral hydronephrosis and 1.057 Kb deletion of both *FOXF1* and the enhancer region.

### 3.4. Case 5

Cystic hygroma was detected in a fetus at 11 weeks of gestation. Chorionic villus sampling identified a normal female karyotype (46,XX), while microarray analysis revealed a 1.171 Mb microdeletion on 16q24.1 involving the *FOXF1* gene. The paternal origin of the deletion could not be determined. Subsequent ultrasound scans detected an atrioventricular septal defect, bilateral superior vena cavae, suspected abnormal pulmonary venous return, and bowel atresia. Postnatally, the baby exhibited severe respiratory distress, suggestive of ACDMPV. An echocardiogram confirmed a complex heart malformation, characterized by a partial atrioventricular septal defect, mild atrioventricular valve insufficiency, bilateral superior vena cavae, a mildly hypoplastic distal transverse aortic arch, normal pulmonary venous return, and suprasystemic right ventricular pressure. She passed away at two days of life. A postmortem lung biopsy confirmed the diagnosis of ACDMPV [9].

### 3.5. Case 6

A fetus at 12 weeks of gestation with a nuchal translucency of 3.6 mm and subcutaneous edema was suspected of having a univentricular heart malformation. Chorionic villus sampling was performed to study the karyotype, which was found to be normal (46,XY). Microarray analysis identified an interstitial 1.17 Mb deletion of 16q24.1 encompassing two OMIM morbid genes: *FOXF1* and *IRF8*. FISH analysis on the peripheral blood of the parents revealed that the deletion occurred de novo in the fetus. The pregnancy was subsequently terminated at 14 weeks of gestation. External fetal examination did not reveal facial dysmorphism. Autopsy showed severe hypoplastic left heart syndrome associated with mitral stenosis and aortic atresia. The kidneys also exhibited rare tubular and glomerular microdilations and, in the lungs, some misaligned pulmonary veins adjacent to pulmonary arteries and bronchioles were observed [10].

### 3.6. Case 7

Wang et al. [11] described the pregnancy of a healthy woman with no family history of congenital malformations and no consanguinity between the parents. Prenatal ultrasound at 23 + 5 weeks of gestation revealed multiple malformations, including pulmonary artery dilatation, complete atrioventricular septal defect, a common atrioventricular valve (CAV), foramen ovale closure, atrial septal defect, ventricular septal defect, and right heart enlargement. Additional anomalies identified included stomach dilatation, esophageal dilation, likely due to pyloric obstruction and a hypodense mass in the upper pole of the left kidney. Karyotype analysis on amniotic fluid was normal (46,XX), while microarray analysis revealed a 2.12 Mb pathogenic microdeletion in the 16q24.1 region, encompassing *FOXF1*, *FOXC2* and other regulatory genes. The microdeletion was confirmed to have occurred de novo. The pregnancy was subsequently terminated.

### 3.7. Case 8

This was the third pregnancy of non-consanguineous parents with no family history of congenital malformations. A high risk of trisomy 21 (1:108) was detected through a non-invasive serum screening test. The measurement of nuchal translucency was 2.4 mm (<95th percentile) with a CRL of 71.5 mm. At 20 weeks of gestation, ultrasound revealed bilateral pyelectasis. Amniocentesis was performed and microarray analysis identified a 0.74 Mb deletion in the 16q24.1 region, encompassing *FOXF1* and other OMIM genes: *FOXC2*, *FOXL1*, *FENDRR*, *MTHFSD*, *LINC01081*, and *LINC01082*. Analysis of parental blood revealed that the deletion occurred de novo on maternal chromosome 16. At 28 weeks of gestation, the fetus developed hydrops and placental hypertrophy. Labor was induced at 34 weeks of gestation. A male infant was born with an Apgar score of 1, presenting with severe respiratory distress consistent with ACDMPV and was placed under palliative care. Postmortem examination revealed right foot contracture, generalized edema, and fluid accumulation in the pleural and peritoneal cavities. The kidneys exhibited characteristics of renal dysplasia and histopathological findings in the lungs were consistent with the spectrum of ACDMPV [5].

### 3.8. Case 9

**Figure 1:**
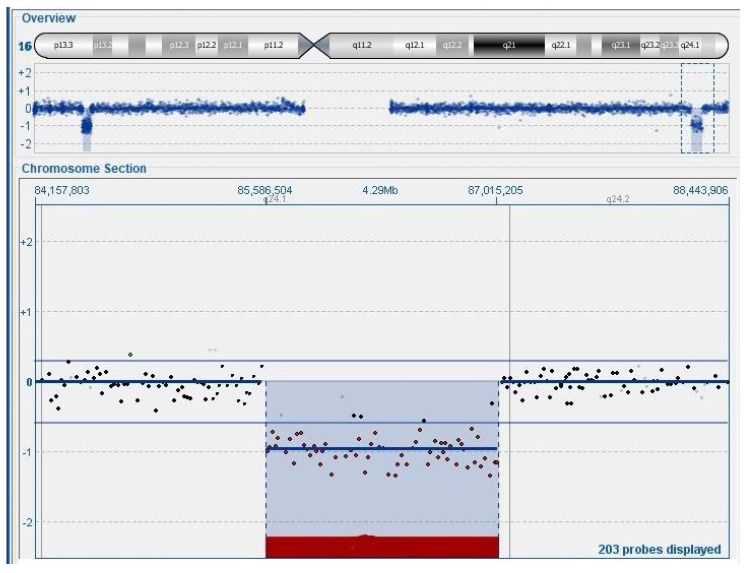
Molecular cytogenetic analyses: array-CGH profile of chromosome 16 of case 9. The red bar highlights the region of the detected deletion.

**Figure 2:**
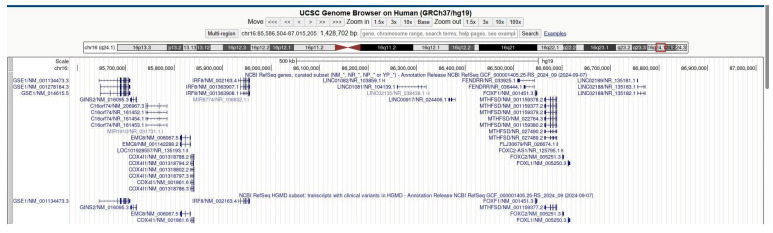
Physical map of the 16q24.1 region (nucleotides 85,586,504 to 87,015,205, GRCh37/hg19) adapted from UCSC Genome Browser, region involved in the microdeletions of case 9. All coding genes included in this region are annotated.

In our center, we identified a fetus at 11 + 5 weeks of gestation with high risk of trisomy 21 (1:4) through non-invasive serum screening test and nuchal translucency of 8 mm. Chorionic villus sampling was performed to study the karyotype, which was found to be normal (46,XX). Microarray analysis identified two deletions on chromosome 16: the first, approximately 1.08 Mb on 16p13.3, and the second, approximately 1.43 Mb on 16q24.1 (Figure 1), involving the *FOXF1* and other genes: *GSE1*, *GINS2*, *C16orf74*, *MIR1910*, *EMC8*, *COX4I1*, *IRF8*, *MIR6774*, *LINC01082*, *LINC01081*, *LINC02135*, *LINC00917*, *FENDRR*, *MTHFSD*, *FOXC2*-*AS1*, *FOXC2,* and *FOXL1* genes (Figure 2). Analysis of the peripheral blood of the parents revealed that the deletions occurred de novo in the fetus. Parental karyotype analysis excluded any structural chromosomal rearrangements in the parents. The pregnancy was subsequently terminated at 14 weeks of gestation. Unfortunately, we could not analyze if the deletion occurs in the maternal or paternal chromosome 16, due to the lack of available material.

## 4. Discussion

We identified nine prenatal cases with a deletion in the 16q24.1 region, all of which involved the *FOXF1* gene or enhancer region. According to the literature, the main ultrasound findings emerged from about 20 weeks of gestation onward and predominantly included cardiac, renal, and intestinal malformations. In cases detected earlier (around 11–12 weeks of gestation), the primary ultrasound findings were increased nuchal translucency and cystic hygroma. In most cases where the pregnancy progressed to birth, the infants developed ACDMPV and passed away within a few days. Table 1 summarizes the main clinical and genetic features of the nine 16q24.1 deletion cases reported, with the aim of providing a comprehensive overview of the data.

*FOXF1* is a member of the Forkhead Box (FOX) transcription factor superfamily and plays a crucial role in cellular proliferation and differentiation. In particular, it is essential for the proper development of the lungs, as well as the gastrointestinal and urinary tracts.

The significance of *FOXF1* in the pathogenesis of alveolar capillary dysplasia with misalignment of pulmonary veins (ACDMPV) was first proposed by Stankiewicz et al. in 2009 [4]. Their research identified recurrent 16q24.1 deletions and point mutations in *FOXF1* in several patients diagnosed with ACDMPV, strongly suggesting that *FOXF1* plays a fundamental role in the disease.

Complete knockout mice (Foxf1 −/−) do not survive embryonic development due to severe mesodermal and vascularization defects, confirming the gene’s essential role. In contrast, heterozygous mice (Foxf1 +/−), which have only one functional copy of the gene, survive until birth but display pulmonary and gastrointestinal malformations similar to those observed in human patients with ACDMPV. These findings suggest that *FOXF1* haploinsufficiency is a key mechanism underlying lung malformations [12].

Studies on mouse embryos have demonstrated that *FOXF1* is already expressed in mesenchymal lung tissue, where it is regulated by the Sonic Hedgehog (SHH) signaling pathway, which is essential for pulmonary development. *FOXF1*, in turn, regulates the expression of several downstream genes, such as those involved in the Notch pathway, as well as endothelial and collagen genes. These are implicated in lung development, angiogenesis, and are thought to contribute to pulmonary hypertension, pulmonary fibrosis, and respiratory dysfunction. The interaction between *FOXF1*-SHH and other membrane proteins, such as semaphorins-neuropilin and vascular endothelial growth factors/vascular endothelial growth factor receptor 2 (VEGF/VEGFR2), is also likely responsible for structural anomalies affecting multiple organ systems, including the heart, gastrointestinal tract, and urinary system. This complex interplay may contribute to the severe malformations observed in association with ACDMPV and suggests that *FOXF1* does not act alone but likely interacts with additional signaling pathways in the disease’s pathogenesis [2,4,7,11].

In addition, the 16q24.1 chromosomal region, where *FOXF1* is located, contains other genes belonging to the Forkhead transcription factor cluster, such as *FOXC2* and *FOXL1*, which are frequently involved in deletions associated with ACDMPV. It is likely that interactions between the deleted genes influence disease severity, particularly the interplay between *FOXF1*, *FOXC2*, *FOXL1*, and *FENDRR* [11].

Interestingly, *FOXC2* has been identified as the key gene responsible for Lymphedema-Distichiasis Syndrome (LDS, MIM 153400), a condition characterized by limb lymphedema and a double row of eyelashes. Mouse models with *FOXC2* inactivation exhibit ventricular septal defects (VSD) and interrupted aortic arch. This finding may explain the cardiac malformations frequently observed in our revised prenatal cases. Meanwhile, *FENDRR* is known to regulate *FOXF1* expression both in cis and trans, supporting the hypothesis that it may contribute to ACDMPV pathogenesis [11]. Fendrr (−/−) mice died either during pregnancy due to heart and body wall defects or in the perinatal period due to malformations of the lungs, heart, and gastrointestinal tract [11].

A crucial discovery in many ACDMPV cases is the presence of overlapping deletions upstream of *FOXF1*, affecting regulatory regions without directly involving the *FOXF1* gene itself. This suggests that these upstream sequences contain important regulatory elements that influence *FOXF1* expression. Among them, researchers have identified a 60 kb non-coding, evolutionarily conserved enhancer region, located approximately 272 kb upstream of *FOXF1* [8,11,13,14].

This enhancer region plays a direct role in regulating *FOXF1* expression by physically interacting with its promoter. It also harbors lung-specific non-coding RNA (lncRNA) genes, such as *LINC01081*, which has been shown to positively regulate *FOXF1* expression [8]. Interestingly, this region, in addition to containing the *FENDRR* gene, also includes *HOXA13* [11], which has been shown to be essential for placental maturation in mice and for regulating Foxf1 expression.

In the prenatal cases we reviewed, some clinical variability was observed. To date, no clear genotype–phenotype correlation or phenotypic differences between *FOXF1* deletions and enhancer region deletions have been reported. It is hypothesized that this variability may be related to *FOXF1* expression levels, which, in turn, could be influenced by the presence of “modifier genes” [2].

Additionally, this region exhibits differential methylation patterns, suggesting epigenetic regulation. Notably, it contains GLI2 binding sites that overlap with a differentially methylated CpG island within the intronic region of another lncRNA, *LINC01082* [8]. These findings further support the idea that *FOXF1* expression is tightly regulated by both genetic and epigenetic mechanisms and that disruptions in this regulatory network may contribute to ACDMPV.

Further evidence supporting the imprinting of *FOXF1* comes from the observation that the 16q24.1 deletion, found in half of ACDMPV patients, occurs de novo and predominantly affects the maternal chromosome [8,13,14,15], suggesting that *FOXF1* is partially paternally imprinted on the human lung.

It is well known that chromosome 16 is subject to imprinting mechanisms. Maternal uniparental disomy (UPD16) is one of the most frequently reported forms of UPD and is often associated with confined placental mosaicism with trisomy 16.

Maternal UPD16 has been linked to intrauterine growth restriction (IUGR), congenital heart defects, and other malformations, whereas paternal UPD16 is generally associated with a normal phenotype or, at most, isolated IUGR [13].

Epigenetic regulation may explain the differences between maternal and paternal UPD(16). Szafranski et al. (2016) [9] proposed two distinct imprinting models for *FOXF1*: the paternal imprinting model, in which *FOXF1* is active on the maternal allele and inactive on the paternal allele, and the maternal imprinting model, in which the activation pattern of *FOXF1* is reversed.

In the paternal imprinting model, deletion of the maternal enhancer region leads to ACDMPV, whereas deletion of the paternal enhancer is benign, as the maternal chromosome remains active.

In contrast, in the maternal imprinting model, deletion of the paternal enhancer significantly reduces *FOXF1* expression, resulting in embryonic lethality. Deletion of the maternal enhancer also decreases *FOXF1* expression, leading to ACDMPV.

This analysis suggests that the paternal imprinting model is more likely. Supporting this hypothesis is the fact that multiple congenital malformations—such as heart defects and pulmonary hypoplasia are observed in maternal UPD(16), closely resembling ACDMPV, whereas paternal UPD(16) is generally associated with a milder phenotype.

The involvement of *FOXF1* in tumor pathogenesis further supports the role of epigenetic mechanisms in cancer. In invasive ductal carcinomas of the breast, *FOXF1* is frequently hypermethylated and consequently silenced, suggesting a potential tumor suppressor function. Conversely, other studies propose that *FOXF1* may act as an oncogene in the metastatic progression of breast cancer [12,16,17]. To date, however, there is no unanimous consensus regarding the precise role of *FOXF1* in cancer development.

## 5. Conclusions

A comprehensive understanding of the molecular and epigenetic mechanisms involving *FOXF1* and the 16q24.1 deletion, together with the recognition of the prenatal ultrasound features associated with ACDMPV, could significantly clarify the etiopathogenesis of the disease. This could improve our knowledge of the disease, enabling specialists to develop better management strategies and provide parents with comprehensive information on prognosis and recurrence risk through informed genetic counseling. Unfortunately, to date, the definitive diagnosis of ACDMPV still depends on histopathological lung examination and, due to the extreme severity and poor prognosis of the condition, is often performed post-mortem. However, the presence of cardiac, renal, and gastrointestinal malformations, as well as increased nuchal translucency/cystic hygroma/fetal hydrops in the first trimester, frequently observed in association with ACDMPV, should raise suspicion and prompt invasive prenatal testing. This includes both fetal karyotyping and array-CGH analysis. These are in accordance with the joint recommendations of the Italian Society of Human Genetics (SIGU) and the Italian Society of Obstetric and Gynecological Ultrasound (SIEOG) (2017). The recommendations are regarding the use of arrays in prenatal diagnosis for ultrasound-detected malformations, particularly when anomalies are nonspecific and not suggestive of a specific genetic condition. The use of next-generation sequencing (NGS) techniques is not currently included in these recommendations, but it may be considered when clinically indicated. Early identification of a deletion involving the *FOXF1* gene is crucial for planning appropriate perinatal clinical management. It also empowers parents to make informed decisions based on their individual context and prepares them for the possible evolution of the disease.

The hypothesis that *FOXF1* is regulated by epigenetic mechanisms is a crucial element that could significantly impact the estimated recurrence risk of ACDMPV. If epigenetic mechanisms are indeed involved, the recurrence risk may be higher than that observed in other cases caused by de novo microdeletions. Further research is therefore essential to clarify this aspect and improve genetic counseling.

## Figures and Tables

**Table 1:** Summary of reported prenatal cases with 16q24.1 deletion involving the *FOXF1* gene. Gestational weeks (GW) at the time of diagnosis, ultrasound (US), alveolar capillary dysplasia with misalignment of pulmonary veins (ACDMPV), unknown (UNK), and common atrioventricular valve (CAV). The letter X indicates that the *FOXF1* gene/Enhancer region is involved in the deletion.

Case	GW	US Findings	Postnatal Investigation	Postnatal Findings	Size	*FOXF1*	Enhancer Region	Other OMIM Genes
1	18	Cystic hygroma, fetal hydrops, single umbilical artery	Dysmorphologic evaluation after death	Low-set ears and edema of the soft tissues in the neck	1.1 Mb	X	X	*MTHFSD*, *FOXL1*, *IRF8*, *COX4I1*, *COX4NB*
2	20	Atrioventricular septal defect, dilated bowel loops	Dysmorphologic evaluation after death, postnatal autopsy, Histopathological assessment of postmortem lung tissue	Atrioventricular septal defects, dilated bowel loops, duodenal atresia, ACDMPV	-	X	X	*IRF8*
3	UNK	Cystyic hygroma and hydronefrosis, single umbilical artery and aortic coartation	UNK	UNK	1.596 Kb	X	X	*COX4I1*, *EMC8*, *FENDRR*, *FOXC2*, *FOXL1*, *IRF8*, *LINC01081*, *LINC01082*, *MTHFSD*
4	UNK	Bilateral hydronefrosis	UNK	UNK	1.057 Kb	X	X	*FENDRR*, *FOXC2*, *FOXL1*, *LINC01081*, *LINC01082*, *MTHFSD*
5	11	Cystic hygroma, atrioventricular septal defect, Bilateral superior vena cavae, suspected abnormal pulmonary venous return, bowel atresia	Postnatal echocardiogram, lung histologic exam on chest autopsy	Respiratory distress, partial atrioventricular septal defect, mild atrioventricular valve insufficiency, Bilateral superior vena cavae, hypoplastic aortic arch, ACDMPV	1.171 Mb	X		*FENDRR*, *FOXC2*, *FOXL1*, *LINC01081*, *LINC01082*, *MTHFSD*
6	12	Increased fetal nuchal translucency (3.6 mm), subcutaneous edema, suspected univentricular heart malformation	Fetal autopsy, histopathological assessment of postmortem lung and kidney tissue	Hypoplastic left heart syndrome, mitral stenosis and aortic atresia, tubular and glomerular microdilatation, ACDMPV	1.17 Mb	X	X	*COX4I1*, *EMC8*, *FENDRR*, *GINS2*, *GSE1*, *IRF8*, *LINC01081*, *LINC01082*, *MTHFSD*
7	23 + 5	Pulmonary artery dilatation, complete atrioventricular septal defect, CAV, foramen ovale closure, atrial septal defect, ventricular septal defect, right heart enlargement, pyloric obstruction, kidney hypodense mass	UNK	UNK	2.12 Mb	X		*COX4I1*, *EMC8*, *FENDRR*, *FOXC2*, *FOXL1*, *GINS2*, *GSE1*, *IRF8*, *LINC01081*, *LINC01082*, *MTHFSD*
8	20	Bilateral pyelectasis, Hydrops, placental hypertrophy	Fetal autopsy, histopathological assessment of postmortem lung and kidney tissue	Respiratory distress, ACDMPV, right foot contracture, generalized edema, pleural and peritoneal fluid accumulation, renal dysplasia	0.74 Mb	X		*FENDRR*, *FOXC2*, *FOXL1*, *LINC01081*, *LINC01082*, *MTHFSD*
9	11 + 5	increased fetal nuchal translucency (8 mm)	Not performed	UNK	1.43 Mb	X		*COX4I1*, *EMC8*, *FENDRR*, *FOXC2*, *FOXL1*, *GINS2*, *GSE1*, *IRF8*, *LINC01081*, *LINC01082*, *MTHFSD*

## Data Availability

The data presented in this study are available on request to the corresponding author.
